# Analysis of transcriptional response in haploid and diploid *Schizosaccharomyces pombe* under genotoxic stress

**DOI:** 10.1093/g3journal/jkae177

**Published:** 2024-08-09

**Authors:** Joshua M Park, Susan L Forsburg

**Affiliations:** Section of Molecular & Computational Biology, University of Southern California, 1050 Childs Way, RRI 108, Los Angeles, CA 90089, USA; Section of Molecular & Computational Biology, University of Southern California, 1050 Childs Way, RRI 108, Los Angeles, CA 90089, USA

**Keywords:** fission yeast, haploid, diploid, RNA-seq, transcriptome, MMS

## Abstract

Whole genome duplications are implicated in genome instability and tumorigenesis. Human and yeast polyploids exhibit increased replication stress and chromosomal instability, both hallmarks of cancer. In this study, we investigate the transcriptional response of *Schizosaccharomyces pombe* to increased ploidy generally, and in response to treatment with the genotoxin methyl methanesulfonate (MMS). We find that treatment of MMS induces upregulation of genes involved in general response to genotoxins, in addition to cell cycle regulatory genes. Downregulated genes are enriched in transport and sexual reproductive pathways. We find that the diploid response to MMS is muted compared to the haploid response, although the enriched pathways remain largely the same. Overall, our data suggests that the global *S. pombe* transcriptome doubles in response to increased ploidy but undergoes modest transcriptional changes in both unperturbed and genotoxic stress conditions.

## Teaser

In this study, Park and Forsburg investigate the role of ploidy on the *S. pombe* transcriptome in unperturbed conditions and in response to genotoxic stress. The authors find that the diploid response to MMS is muted compared to the haploid response, although the enriched pathways remain largely the same.

## Introduction

Recent studies have implicated whole genome duplications (WGD), or polyploidy, as a driver of genome instability and tumorigenesis in higher eukaryotic models (Fujiwara *et al.* 2005; Davoli and de Lange 2011; Gemble *et al.* 2022). Polyploid cells exhibit increased replication stress and DNA damage (Nano *et al.* 2019; Gemble *et al.* 2022) as well as mitotic defects leading to chromosomal instability (CIN) and aneuploidy (Dewhurst *et al.* 2014; Kuznetsova *et al.* 2015). Although research in higher eukaryotic models is valuable, there are challenges in studying polyploidy. Tetraploidy has been shown to induce a p53-mediated G1 arrest (Ganem *et al.* 2014) and analysis of tumors found a correlation between WGD and p53 disruption (Zack *et al.* 2013; Quinton *et al.* 2021). Polyploid cells that maintain wild-type p53 may have other factors that allow evasion of the checkpoint and tolerance of higher ploidy (Crockford *et al.* 2017; Zeng *et al.* 2023).

To avoid these challenges, yeast has been used as a model organism to study polyploidy. *Saccharomyces cerevisiae* and *Schizosaccharomyces pombe* have been shown to tolerate multiple levels of WGD (Mortimer 1958; Molnar and Sipiczki 1993). Tetraploids in *S. cerevisiae* exhibit genome instability such as increased chromosome loss, DNA damage, and sensitivity to genotoxic stress (Storchová *et al.* 2006). The phenotypes are similar to those observed in higher eukaryotic models, which suggests that yeast is a suitable model to study the effects of WGDs on genome stability. While *S. cerevisiae* has been used extensively to study polyploidy (reviewed in Storchova 2014), *S. pombe* studies have been lacking. The *S. pombe* genome has pathways in genome organization, maintenance, and repair that are conserved in higher eukaryotes (reviewed in Hoffman 2015). *S. pombe* retains complex centromeres, H3K9-mediated heterochromatin, and other conserved elements that are lost in *S. cerevisiae*, providing a compelling reason to study WGD in *S. pombe* as well.

In this study, we use messenger RNA (mRNA)-seq on haploid and diploid *S. pombe* to investigate any changes in the transcriptome when the genome is doubled. We also investigated the transcriptional response of the cells to methyl methanesulfonate (MMS) treatment in both haploid and diploids and observed an increase in expression for genes involved in genotoxin response and a decrease in expression for genes involved in transport and reproduction. The diploid response to MMS is dampened but reflects similar pathways as haploids. Taken together, we observe that *S. pombe* diploids have modest changes in transcription profile compared to haploids when unperturbed and in response to the genotoxin MMS.

## Materials and methods

### Yeast strains and gowth

*S. pombe* strains used are listed in Supplementary Table 1. *S. pombe* cells were cultured following standard protocols and methods (Forsburg and Rhind 2006). The use of the *smt-0* strain prevents mating-type switching and formation of a double-strand break at the *mat* loci (Engelke *et al.* 1987). The smt-0 mutation which abolishes mating-type switching in fission yeast is a deletion (Styrkársdóttir *et al.* 1993).

### Diploid generation

Mating-type homozygous diploids were generated by protoplast fusion methods adapted from Ekwall and Thon (2017) and Flor-Parra *et al.* (2014). h^−^*smt-0* mating-type strains with *ade6-M210* and *ade6-M216* complementary alleles were grown separately in 50 mL yeast extract with supplements (YES) media to mid log phase. Cultures were centrifuged at 800 *g* (5 min) and supernatant was discarded. Pellets were washed in 10 mL 0.65 M KCl twice, then resuspended in 2 mL 0.65 M KCl, 0.1 g/mL Lallzyme MMX (Lallemand, supplied by Scott Laboratories). Cells were incubated at 36°C until > 90% protoplast formation (20 min). 20 mL of 10 mM Tris, 1.2 M Sorbitol, pH 7.6 (TS) buffer was added to stop the reaction and cells were centrifuged at 200 *g* (5 minutes). Pellets were washed twice in TS buffer. Pellets were resuspended in 500 µL of 10 mM Tris, 1.2 M Sorbitol, 10 mM CaCl_2_, pH 7.6 (TSC) buffer, mixed together (total 1 mL resuspension), and transferred to 1.5 mL centrifuge tube. Cells were centrifuged at 310 *g* (5 min) and supernatant was discarded. The pellet was resuspended in 1 mL PEG, CaCl_2_ solution (900 µL 30% PEG 6000, 100 µL 0.1 M CaCl_2_) with gentle flicking. Cells were incubated at room temperature for 30 min, then centrifuged at 300 *g* (5 min) and supernatant was discarded. Pellets were resuspended in 500 µL TSC buffer and 10 µL aliquots were plated onto adenine dropout minimal media plates supplemented with 1.2 M sorbitol. Diploid genome content was validated by flow cytometry (BD Accuri C6 plus).

### Sample preparation and RNA extraction

Haploid and diploid yeast strains were cultured overnight in 50 mL YES media to mid log phase. Cultures were split in half into no treatment and 0.0075% MMS (Sigma-Aldrich) treated groups and cultured for an additional 4 h. Cells were counted on a Bright-Line Hemacytometer (Hausser Scientific), and the same number of cells were harvested for each sample (1.5 × 10^8^ cells). Samples were washed once in Milli-Q water before processing. Total RNA was extracted using the RNeasy Mini Kit (Qiagen) with the addition of on-column DNase treatment (RNase-Free DNase Set, Qiagen). RNA was further treated with Turbo DNase (Invitrogen) to remove excess genomic DNA. ERCC RNA Spike-in Mix (Invitrogen) was added to the samples as an external control. Mix 1 was added to the haploid and diploid samples at equal dilution and volumes according to ERCC protocol.

### RNA sequencing and analysis

Total RNA was sent to Novogene Co. Inc. for library preparation and mRNA sequencing. Samples were purified for mRNA by polyA capture and paired-end sequenced on an Illumina platform. Raw reads were aligned to the *S. pombe* annotated reference genome (Pombase) using STAR 2.7.0e (Dobin *et al*. 2013; Dobin and Gingeras 2016). Mapped reads were assigned to genomic features and read counts were summarized into a table for downstream applications using featureCounts (Subread; Liao *et al.* 2014). Read counts were analyzed on R 4.1.1, with the package DESeq2 (Love *et al.* 2014). Global expression changes were analyzed by estimating size factors using 74 ERCC spike-ins as control genes. Differential gene expression analysis was performed using default parameters. Two transcripts were manually filtered out (SPAC977.09c and SPBC1348.10c) because they have the same open reading frame sequence (at different genomic loci) which resulted in a skewed assignment of the reads. Statistical analysis of gene list overlap was done using hypergeometric distribution.

The package AnnotationForge (Carlson and Pagès 2023) was used to generate the *S. pombe* org.db from the NCBI database. Gene ontology (GO) enrichment analysis was performed using clusterProfiler (Yu *et al.* 2012) on the subset of genes that were considered differentially expressed. Benjamini–Hochberg *P*-value adjustment and a *P*-value and *q*-value cutoff of 0.05 parameters were used. Plots were generated using R package enrichplot (Yu 2023).

### Reverse transcription-quantitative PCR validation

Total RNA for RT-qPCR was sampled from the same extractions that were sent for RNA sequencing. Total RNA was reverse transcribed with SuperScript III (Invitrogen) using the oligo dT and random hexamer protocol for mRNA and rRNA targets, respectively. Equal amounts of RNA per sample were processed. Five genes and one ERCC transcript were chosen as representatives for upregulated and downregulated, nondifferentially expressed, and control genes. Additionally, 5 and 18 s rRNA were assayed. qPCR was performed using iTaq Universal SYBR Green Supermix (Bio-Rad) on the CFX96 Connect Real-Time PCR System (Bio-Rad). The 2^−ΔΔCt^ method (Livak and Schmittgen 2001) normalizing to ERCC transcript was used to compare expression levels.

## Results

### Overview of gene expression workflow

To investigate the effect of ploidy on the transcriptome without confounding effects from mating type, we generated a mating-type homozygous diploid *S. pombe* strain by protoplast fusion. We fused 2 heterothallic h^−^*smt-0* haploid strains that are completely isogenic except at the *ade6* locus (*ade6-M210* and *ade6-M216* are used for selection of diploids by intragenic complementation). To assess the change in transcriptome in response to genotoxic stress, we cultured the strains in the absence or presence of 0.0075% MMS. Three biological replicates for each condition were processed for mRNA-seq. We used external RNA spike-ins from ERCC to control for potential changes in global expression levels (Lovén *et al.* 2012) as ploidy doubled.

We obtained an average of 49.6 million reads (24.8 paired-end reads) per sample (Table 1). On average, 91% of the reads were uniquely mapped onto the *S. pombe* reference genome, and 89% of mapped reads were assigned to genes. There were 4,641 *S. pombe* genes analyzed (not including ERCC transcripts) after removing low-expressing genes (fewer than five reads in 2 or more samples).

**Table 1.** Overview of RNA-seq data analyzed in this study.

Strain	Raw reads (PE) (M)	Uniquely mapped reads	Reads assigned
Haploid NT 01	21.6	19.9 M (92%)	17.7 M (89%)
Haploid NT 02	19.7	18.3 M (93%)	16.3 M (89%)
Haploid NT 03	20.6	18.9 M (92%)	16.8 M (89%)
Haploid MMS 01	25.6	23.5 M (92%)	21.1 M (90%)
Haploid MMS 02	26.5	24.3 M (92%)	21.8 M (90%)
Haploid MMS 03	32.0	29.5 M (92%)	26.5 M (90%)
Diploid NT 01	24.2	21.4 M (88%)	19.1 M (89%)
Diploid NT 02	20.6	18.6 M (90%)	16.6 M (89%)
Diploid NT 03	21.2	19.2 M (91%)	17.1 M (89%)
Diploid MMS 01	32.2	29.4 M (91%)	26.4 M (90%)
Diploid MMS 02	22.2	20.2 M (91%)	18.1 M (90%)
Diploid MMS 03	31.2	28.3 M (91%)	24.9 M (88%)

Three biological replicates for each experimental group denoted by 01, 02, and 03. Raw paired-end reads (PE) and reads retained after each step of the mapping pipeline are listed.

### Global and differential gene expression of haploid and diploid *S. pombe*

We first compared global changes in the transcriptome of diploids compared to haploids. We observed that the total RNA extracted from the diploid samples were approximately twice the amount compared to the haploid samples, both in the untreated samples and those treated with 0.0075% MMS, when the same number of cells were processed (Supplementary Fig. 1a). We then compared the mRNA abundance by comparing the fragment per kilobase million of each transcript that was normalized to the external spike-ins. We found that, across 4,641 transcripts, expression was increased by 1.9-fold on average in the diploid compared to the haploid samples (Supplementary Fig. 1b). We validated these results by probing 5 genes (2 upregulated, 2 no change, and 1 downregulated) by RT-qPCR normalized to an ERCC spike-in transcript (Supplementary Fig. 1c). We also assessed the abundance of rRNA transcripts by RT-qPCR. We targeted 5s and 18 s rRNA which are RNA polymerase III and I targets, respectively. We observed that the 5 and 18 s transcripts were increased by 1.6- and 1.9-fold, respectively, in diploid samples compared to haploids (Supplementary Fig. 1d). Taken together, these data suggest that the global transcriptome in *S. pombe* diploids is approximately doubled compared to haploids.

**Fig. 1.**
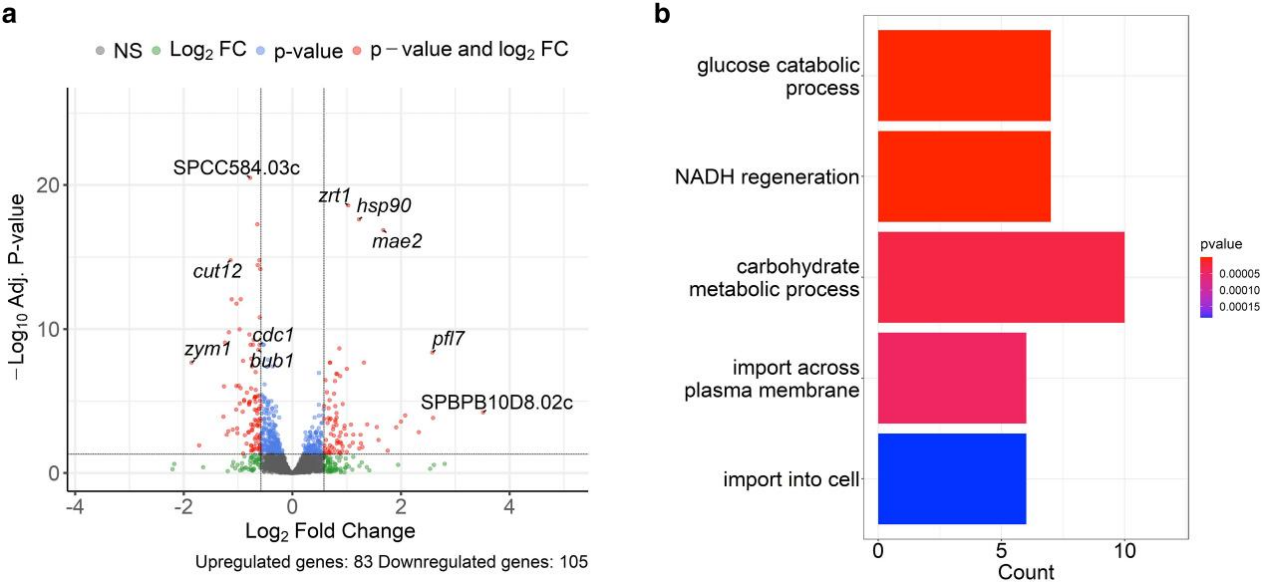
Diploids have increased expression for cell membrane-associated genes. a) Volcano plot displaying differential gene expression in diploid *S. pombe* compared to haploids. Genes that are logFC > 0.58 or < (− 0.58) (fold change ± 1.5) are to the left and right of the vertical lines (labeled green) and adjusted *P*-value < 0.05 are above the horizontal line (labeled blue). Genes that satisfy both thresholds are in the top left and top right quadrants (labeled red) and are considered differentially expressed. b) Bar plot displaying enriched GO terms for biological processes in the upregulated DEGs. The *x*-axis represents the number of genes that contain the specific biological process term.

We performed a pairwise comparison of gene expression between haploid and diploid *S. pombe* in normal vegetative growth, to assess whether increased ploidy induced differential expression. There were a total of 188 differentially expressed genes (DEGs; 1.5-fold change, adjusted *P*-value < 0.05): 83 upregulated and 105 downregulated (Fig. 1a). The upregulated genes were enriched for GO terms related to metabolic processes and transport across the cell membrane (Fig. 1b). These genes were also enriched for localization at the plasma membrane/cell surface (Supplementary Fig. 2). Despite 105 downregulated genes, there were no enriched GO terms. Interestingly, 2 cell cycle associated genes that were downregulated in the diploids included *cdc1+* (polymerase delta subunit) and *bub1+* (spindle assembly checkpoint kinase) (log_2_FC of −0.6 for both genes; Fig. 1a).

### Differential gene expression after MMS treatment

**Fig. 2.**
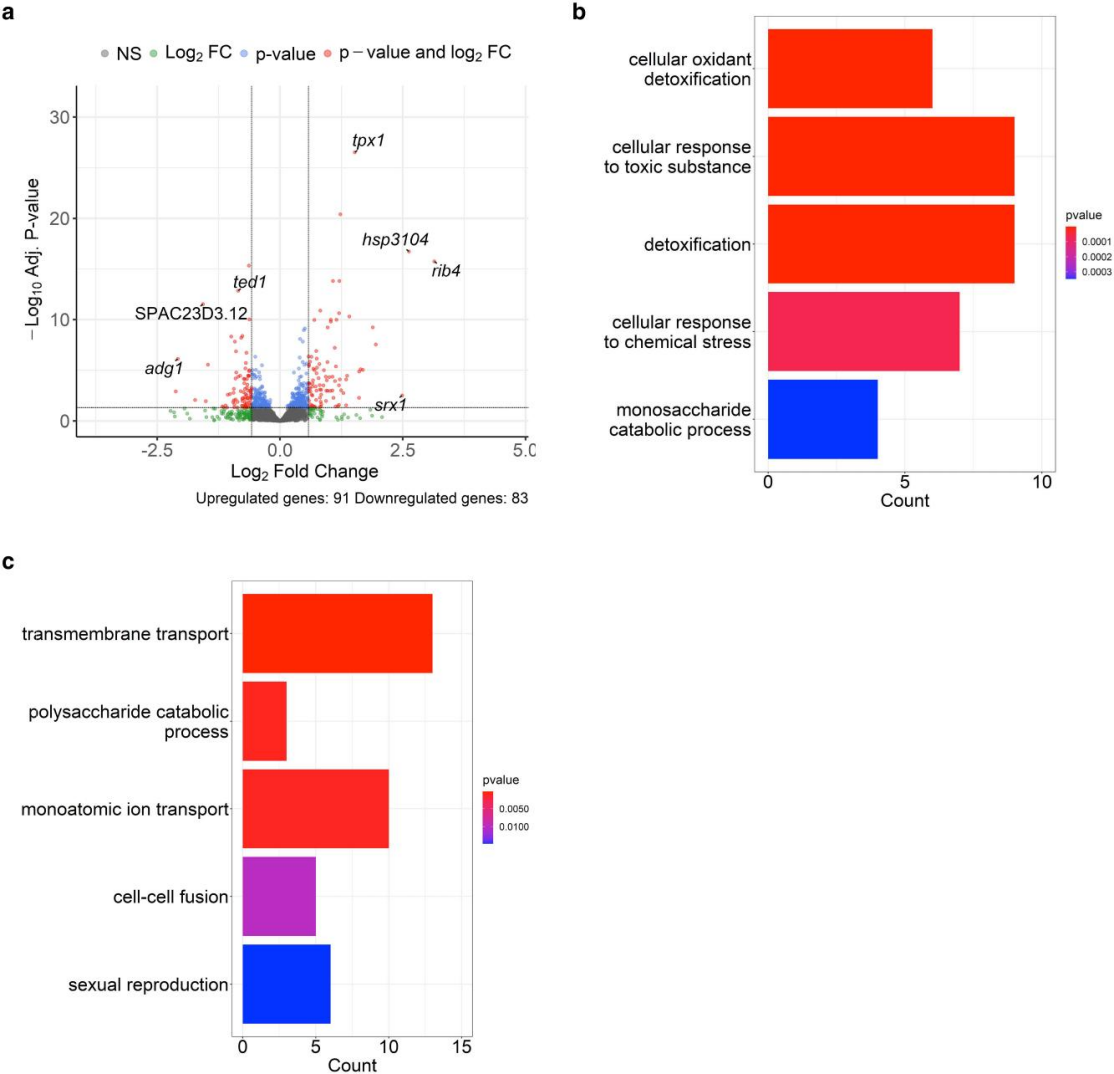
Haploid transcriptional response to MMS. a) Volcano plot displaying differential gene expression between untreated haploids and 0.0075% treated haploids. b) Bar plot displaying enriched GO terms for biological processes in the upregulated DEGs. c) Bar plot displaying enriched GO terms for biological processes in the downregulated DEGs.

**Fig. 3.**
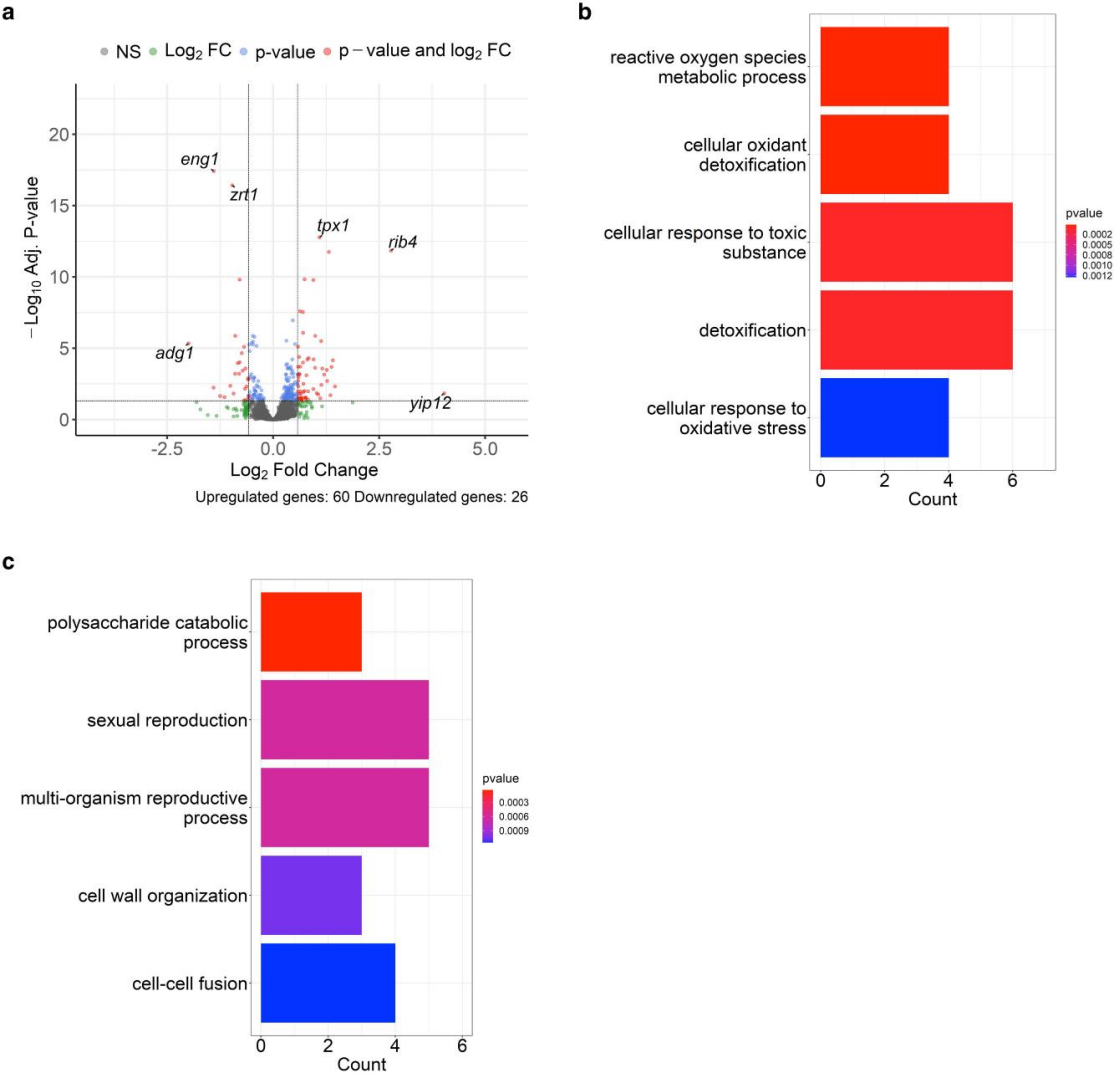
Diploid transcriptional response to MMS. a) Volcano plot displaying differential gene expression between untreated diploids and 0.0075% treated diploids. b) Bar plot displaying enriched GO terms for biological processes in the upregulated DEGs. c) Bar plot displaying enriched GO terms for biological processes in the downregulated DEGs.

We analyzed differential gene expression after 4-h treatment of 0.0075% MMS in haploid and diploid *S. pombe.* This dose and timing are typical for growth and sensitivity assays (e.g. Ranatunga and Forsburg 2016). The thresholds for DEGs were the same as the analysis above. In the haploids, there were a total of 174 DEGs: 91 upregulated and 83 downregulated in MMS treated vs untreated conditions (Fig. 2a). The upregulated genes were enriched for GO terms involved in response to toxic substances (Fig. 2b). Among the upregulated genes were a group of Mlu1binding factor (MBF) target transcripts (Supplementary Table 3). This group of genes has been shown to be upregulated in response to hydroxyurea (HU)-induced replication stress in *S. pombe* (Dutta and Rhind (2008)). This suggests that our treatment of MMS is inducing a similar replication stress response as HU treatment, even after 4 h. Downregulated genes were enriched in processes involved in cellular transport and mating/sexual reproduction (Fig. 2c). This repression of mating signaling has also been observed in *S. cerevisiae* in response to MMS (Panessa *et al.* 2023). In the diploids, there were 86 DEGs: 60 upregulated and 26 downregulated (Fig. 3a). The GO enrichment analysis of the upregulated and downregulated gene sets was similar to the haploid MMS analysis; upregulated genes were enriched for functions relating to response to toxic substances and downregulated genes were enriched for metabolic and reproductive functions (Fig. 3, b and c).

**Fig. 4.**
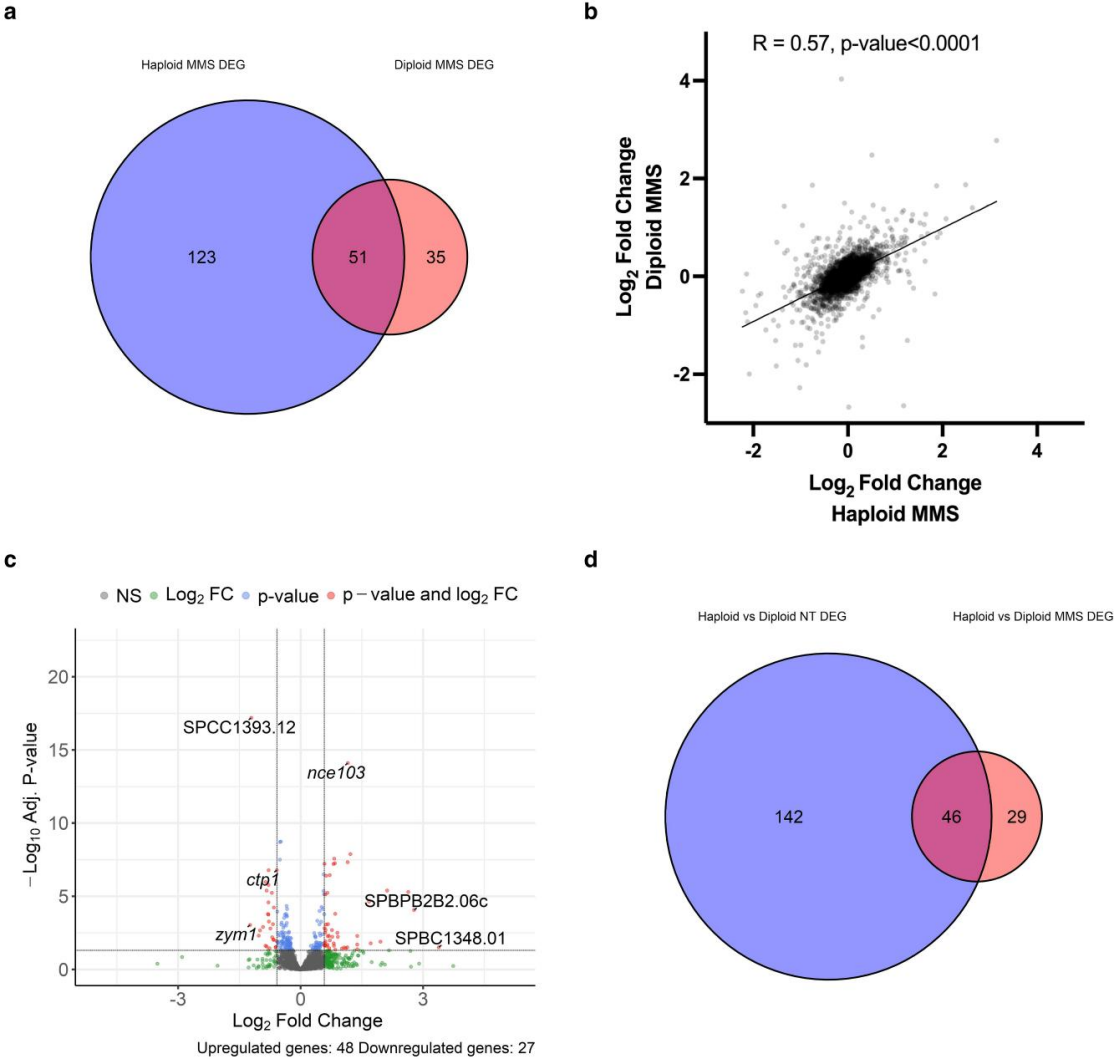
Diploids have a similar transcriptional response to MMS as haploids. a) Venn diagram displaying overlap of DEGs from haploid MMS and diploid MMS analysis (*P*-value of 1.4e – 47). b) Scatter plot displaying a comparison of DEGs in response to MMS across the transcriptome in haploids and diploid. *x*-axis represents logFC of each gene in the haploid dataset, and *y*-axis represents the same for diploids. *R* value of 0.57 is the correlation coefficient. c) Volcano plot displaying differential gene expression between MMS-treated haploids and MMS-treated diploids. d) Venn diagram displaying overlap of DEGs from untreated haploid and diploid analysis compared to the MMS-treated haploid and diploid analysis (*P*-value of 1.1e – 53).

Next, we compared the haploid and diploid response to MMS. There were approximately half as many DEGs in the diploid MMS analysis compared to the haploid: 31 and 57 fewer upregulated and downregulated genes, respectively. When we compared the 174 DEGs from the haploid MMS analysis with the 86 DEGs from the diploid MMS analysis, there were 51 genes that overlapped (Fig. 4a). Genes that were differentially regulated in the haploid MMS analysis but not the diploid MMS analysis included MBF target transcripts, oxidative stress response/detoxification genes, and DNA repair factors (Table 2). When we compared the log-fold change across the transcriptome between the haploid MMS treatment and the diploid MMS treatment analysis, the correlation value was 0.57 (Fig. 4b) suggesting that the diploid response was similar to the haploid on the global scale. We also performed a pairwise comparison of the haploid MMS-treated samples with the diploid MMS-treated samples and found a total of 75 DEGs: 48 upregulated and 27 downregulated (Fig. 4c). Of those 75 DEGs, 46 overlapped with the DEG list from the haploid and diploid no-treatment (NT) analysis (Fig. 4d). This suggests that these 46 DEGs are ploidy-dependent and not affected by MMS treatment, while the other 29 may be ploidy and MMS-dependent DEGs. Interestingly, *ctp1+* (cofactor of Mre11–Rad50–Nbs1 complex, Williams *et al.* 2009) was downregulated in diploids compared to haploids in response to MMS (log_2_FC of −0.58).

**Table 2.** List of DEGs in haploid MMS analysis not observed in diploids.

Gene	Haploid Log_2_-FC; *P*-adj	Diploid Log_2_-FC; *P*-adj
MBF target transcripts		
*cdt1*	2.0; 2.9e – 8	0.9; 0.06
*cdc18*	1.7; 9.2e – 6	0.9; 0.08
*cdc22*	1.2; 3.1e – 4	0.7; 0.1
*tos4*	1.2; 9.8e – 4	0.8; 0.1
*mik1*	1.0; 4.8e – 5	0.2; 0.6
*cig2*	0.8; 1.3e – 2	0.3; 0.07
Oxidative stress response/detoxification		
*trx1*	0.8; 2.5e – 6	0.4; 0.06
*sod2*	0.6; 2.6e – 3	0.4; 0.09
*yhb1*	0.6; 0.04	0.07; 0.9
DNA repair		
*rad54*	0.7; 2.1e – 4	0.4; 0.05
*srs2*	−0.7; 5.6e – 3	−0.1; 0.9

List of notable genes that are differentially regulated in the haploid MMS analysis, but not the diploid MMS analysis [the fold change (± 1.5 fold change) and/or adjusted *P*-value (< 0.05) threshold is not met].

## Discussion

We performed mRNA-seq comparing haploid and mating-type homozygous diploid *S. pombe* in normal conditions and under MMS-induced replication stress. We used h^−^*smt-0* mating-type strains to control for any unwanted effects that imprinting at the mating-type locus may cause (Octobre et al. 2008). Our RNA-seq analysis was performed using external RNA spike-ins to control for possible global changes in the transcriptome due to the doubling of the genome. We find that the WGD results in ∼2-fold increase in the transcriptome including global mRNA expression. Increased mRNA levels have been observed in both *S. pombe* and *S. cerevisiae* as ploidy increases which is correlated with cell size increases (Sun *et al*. 2020; Yahya *et al*. 2022; Swaffer *et al*. 2023).

When analyzing the transcriptome for DEGs in the haploid compared to the diploid, we find that just 188 genes (83 upregulated and 105 downregulated) were differentially regulated out of 4,641 genes observed in the data (Fig. 1a). Our results showed a larger number of DEGs compared to previous research in *S. cerevisiae* in which the investigators found a very small number of genes to be differentially regulated across various levels of polyploidy (Galitski *et al.* 1999; Storchová *et al.* 2006; Yahya *et al.* 2022). However, when we impose more conservative thresholds, the DEGs are in line with *S. cerevisiae* observations (e.g. 2-fold change in expression threshold results in 20 upregulated and 15 downregulated genes). Despite the differences, the upregulated genes in those studies were enriched for cell membrane-associated proteins similar to our *S. pombe* results (Fig. 1b and Supplementary Fig. 1). In both yeast, cell size increases as ploidy increases (Mundker 1953; Wood and Nurse 2015) which may explain the enrichment of cell membrane-associated genes.

Although there were no enriched GO terms for the downregulated genes, we noted 2 cell cycle-associated genes, *cdc1 +* and *bub1 +* (Fig. 1a). Cdc1 is a subunit of polymerase delta that is essential for cell cycle progression, and a mutant strain was previously shown to be hypersensitive to MMS (MacNeill *et al* 1996). Bub1 is a spindle assembly checkpoint kinase that is essential for maintaining diploidy in *S. pombe* (Bernard *et al* 1998). Although the downregulation of these genes does not seem to affect diploid growth in unperturbed conditions, they may play a role in diploid sensitivity to genotoxins discussed below.

A previous report examining the MMS-responsive transcriptome used a microarray protocol, and exposed cells to a high dose for a short period of time to assess the acute response (Chen *et al.* 2003). In our study, we used a lower dose for an extended period, more similar to the conditions used to evaluate MMS sensitivity in growing cells (Mastro and Forsburg 2014; Ranatunga and Forsburg 2016). We found that both MMS-treated haploids and diploids upregulated genes in pathways involved in general response to genotoxins and stress (Figs. 2b and 3b). These genes have functions in oxidation–reduction processes and transmembrane transport. Both processes have been reported to be upregulated in response to MMS in *S. pombe* and other yeasts (Chen *et al.* 2003; Benton *et al.* 2006; Feng *et al.* 2022).

Another set of genes that we observed to be upregulated were MBF-targeted transcripts (Supplementary Table 3). MBF is a cell cycle specific transcription factor that directs expression of S phase genes (Lowndes *et al*. 1992; Ayté *et al.* 2001). MBF-regulated genes have been shown to be upregulated in response to replication stress induced by HU in *S. pombe* (Dutta and Rhind (2008)). These genes are involved in DNA replication and repair pathways necessary for cell survival in response to replication stress (de Bruin *et al.* 2008; Caetano *et al.* 2011). This suggests that our 4-h MMS treatment induces a replication stress response, as expected.

Downregulated genes in MMS treatment were enriched in transport, metabolic, and reproductive processes (Figs. 2c and 3c). Downregulation of transport and metabolic machinery has been observed in *S. pombe* and other yeasts (Chen *et al.* 2003; Benton *et al.* 2006; Feng *et al.* 2022). This indicates a conserved reaction to MMS, presumably to halt the import of the genotoxic agent and redirect resources to respond to the stress. The reproductive genes that were downregulated were predominantly involved in conjugation and entry into meiosis. The downregulation of mating and reproductive processes has similarly been observed in *S. cerevisiae* when treated with MMS (Panessa *et al.* 2023). The link between reproductive processes and genotoxic stress response is not well studied but may be involved in cell cycle dynamics or part of a broad stress response. The downregulation of the mating pathway in response to MMS requires further investigation.

Differential gene analysis of both haploid and diploid *S. pombe* cells in response to MMS showed that diploids have approximately half the number of DEGs compared to haploids in MMS. We observe that several DEGs present in the haploid analysis but not the diploid analysis include MBF-dependent transcripts, oxidative stress response and detoxification genes, and DNA repair factors (Table 2). These genes, in the diploid MMS analysis, follow a similar differential expression pattern but at a smaller magnitude in fold change and/or significance that may not/does not reach statistical significance. However, there was a significant overlap of DEGs and enriched pathways were largely the same (Fig. 4). This suggests that, while the core response to MMS is similar in diploids and haploids, the diploid response is muted. The pairwise comparison between MMS-treated haploids and diploids resulted in 75 DEGs that were not enriched for any specific biological pathway (Fig. 4d). Of these 75 genes, 29 did not overlap with the haploid and diploid NT comparison (Fig. 4d), which suggests their differential expressions are ploidy and MMS treatment dependent. Within this group of 29 genes was Ctp1, a regulator of the Mre11–Rad50–Nbs1 complex that is associated with DNA double-strand break repair (Limbo *et al.* 2007). Although *ctp1 +* expression is upregulated in response to MMS in both haploids and diploids (Supplementary Table 5 and 6), its expression is reduced in diploids compared to haploids in unperturbed (log_2_FC of −0.52, just below the FC threshold) and MMS-treated conditions (log_2_FC of −0.58, Fig. 4c).

In work to be reported elsewhere, we have observed that diploids are more sensitive than haploids to genotoxic stressors such as MMS, HU, camptothecin, phleomycin, X-irradiation, and thiabendazole (JMP and SLF, in preparation). The data presented here suggest that the increased sensitivity to MMS in diploids may be due to a dampened transcriptional response compared to the haploids. Additionally, the reduced expression of key genes such as *cdc1^+^*, *bub1^+^*, or *ctp1^+^* might affect the diploid cell's ability to respond to genome stress. The 29 ploidy and MMS-dependent DEGs may also play a role in the diploid sensitivity and require further investigation.

The data presented in this study provides insight into the transcriptional differences between *S. pombe* haploids and diploids in unperturbed and MMS-treated conditions. Previous studies in budding yeast have looked at changes in transcriptome in response to ploidy changes (Storchová *et al.* 2006; Yahya, *et al.* 2022) or in MMS treatment (Panessa *et al.* 2023). To the best of our knowledge, this is the first study to look at the intersection of ploidy and genotoxic stress at the transcriptome level. As polyploidy has been implicated in genome instability in *S. pombe* and other model systems (Storchová *et al.* 2006; Gemble, *et al*. 2022; JMP and SLF, in preparation), our findings suggest that differential transcriptional responses may provide some explanation. *S. pombe* is therefore a promising model for investigating genome instability in polyploidy.

## Supplementary Material

jkae177_Supplementary_Data

## Data Availability

The data discussed in this publication have been deposited in NCBI's Sequence Read Archive under BioProject ID PRJNA1031691 and Gene Expression Omnibus (Edgar *et al.* 2002) under GEO series accession number GSE271887. Strain list is available in Supplementary Table 1. Primers used for RT-qPCR validation are available in Supplementary Table 2. Full list of RNA-seq results including gene name, log_2_-fold change (logFC) and adjusted *P*-value are available in Supplementary Tables 4–7. All strains and reagents are available upon request. Supplemental material available at G3 online.
