# Supplementary material for: Analysis of transcriptional response in haploid and diploid Schizosaccharomyces pombe under genotoxic stress
Source: G3 (Bethesda). 2024 Aug 9;14(9):jkae177. doi: 10.1093/g3journal/jkae177 (PMC11373635; doi:10.1093/g3journal/jkae177)
Supplement: jkae177_Supplementary_Data [file jkae177_supplementary_data.zip › Supplemental_Material_Legends_G3-2024-405152.pdf]

Figure S1. Global transcriptome is doubled in diploids compared to haploids.

A) Dot plot displaying total RNA extracted from samples for mRNA-seq and RT-qPCR. 3 replicates per condition. B) Scatter plot displaying comparison of log_2 FPKM after spike-in normalization in haploid NT and diploid NT samples averaged across the 3 replicates. C) Bar plot comparing RT-qPCR and RNA-seq fold change for 5 genes (NT condition), normalized to the spike-in ERCC-002 transcript. Dotted horizontal line represents fold change of 1 (no change in expression). D) Dot plot displaying RT-qPCR results of 5s and 18s rRNA transcripts (NT condition).

Figure S2. Upregulated diploid genes are enriched for plasma membrane localization.

Gene-concept network plot displaying enriched cellular compartment terms in the haploid vs diploid no treatment upregulated genes.

Table S4. Haploid vs diploid no treatment DEGs

Table S5. Haploid MMS treatment DEGs

Table S6. Diploid MMS treatment DEGs

Table S7. Haploid MMS vs diploid MMS DEGs
