# Supplementary material for: Analysis of transcriptional response in haploid and diploid Schizosaccharomyces pombe under genotoxic stress
Source: G3 (Bethesda). 2024 Aug 9;14(9):jkae177. doi: 10.1093/g3journal/jkae177 (PMC11373635; doi:10.1093/g3journal/jkae177)
Supplement: jkae177_Supplementary_Data [file jkae177_supplementary_data.zip › Table_S1_G3-2024-405152.pdf]

Table S1	List of strains used in this study	
FY 9699	h- smt-0 his3D-1 ura4-D18 leu1-32 ade6-M210	This study
FY 9786	h- smt-0/h- smt-0 his3D-1/his3D-1 ura4-D18/ura4-D18 leu1-32/leu1-32 ade6-M210/ade6-M216	This study
