# Supplementary material for: Analysis of transcriptional response in haploid and diploid Schizosaccharomyces pombe under genotoxic stress
Source: G3 (Bethesda). 2024 Aug 9;14(9):jkae177. doi: 10.1093/g3journal/jkae177 (PMC11373635; doi:10.1093/g3journal/jkae177)
Supplement: jkae177_Supplementary_Data [file jkae177_supplementary_data.zip › Table_S2_G3-2024-405152.pdf]

Table S2	List of qPCR primers used in this study	
Gene	Forward Primer (5’ to 3’ sequence)	Reverse Primer (5’ to 3’ sequence)
str3	CGACCAAATTTCGCGGCTAC	GGCATGATACCTGAGACCCC
alg9	GAAGGCCACAGTCAACGAAC	TATCGGCCCCATTTAGGCTC
SPAC977.18	GCACGAACCAGCAAACTTGG	GAGATTCGGGACTAGCATCGG
yak3	AGGAGAACTTTGGCGCTCTC	GGGCATAGGAGGGGTATCCA
zym1	TGTAAGAGCAAACAAGGCAAGC	TCGAAGCACATTTGCAAGAAGA
ERCC-002	CTCGGGTCTGCGGGTTATAG	ACCAGTACTGATTGCCGAGC
5s rRNA	CCAGTTCCCGTCCGATCAC	AAAGGAAGCCTACAGCACCC
18s rRNA	AAGCAGGCAAGTTTTGCTCG	TGAATACGAATGCCCCCGAC
