# Supplementary material for: Analysis of transcriptional response in haploid and diploid Schizosaccharomyces pombe under genotoxic stress
Source: G3 (Bethesda). 2024 Aug 9;14(9):jkae177. doi: 10.1093/g3journal/jkae177 (PMC11373635; doi:10.1093/g3journal/jkae177)
Supplement: jkae177_Supplementary_Data [file jkae177_supplementary_data.zip › Table_S3_G3-2024-405152.pdf]

Table S3 List of upregulated MBF Transcripts

**Gene**	**Log_2 Fold Change**	**p-adj.**
ctp1	0.7	1.1e-10
cdt1	2.0	2.9e-8
cdc18	1.7	9.2e-6
cdt2	1.4	3.5e-5
mik1	1.0	4.8e-5
cdc22	1.2	3.1e-4
tos4	1.2	9.8e-4
cig2	0.8	1.3e-2
ssb1	0.4	5.0e-2
mrc1	0.1	4.0e-1

List of MBF target transcripts and their Log_2 fold change values and p-values from the haploid MMS comparison dataset.
