# Supplementary figures and images for: Analysis of transcriptional response in haploid and diploid Schizosaccharomyces pombe under genotoxic stress
Source: G3 (Bethesda). 2024 Aug 9;14(9):jkae177. doi: 10.1093/g3journal/jkae177 (PMC11373635; doi:10.1093/g3journal/jkae177)

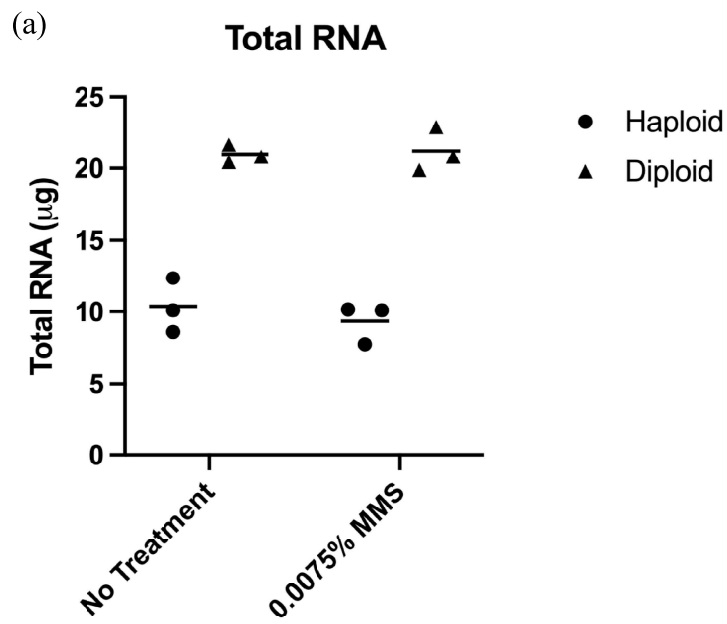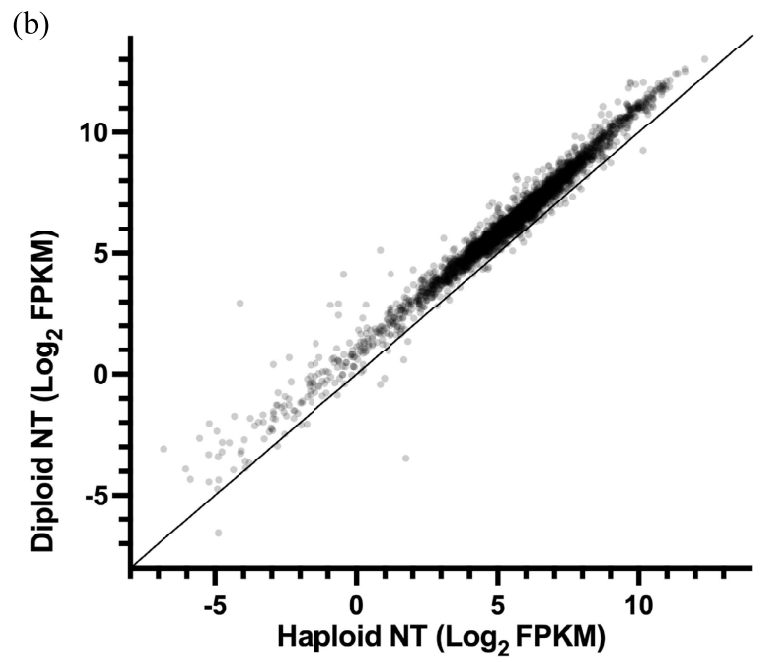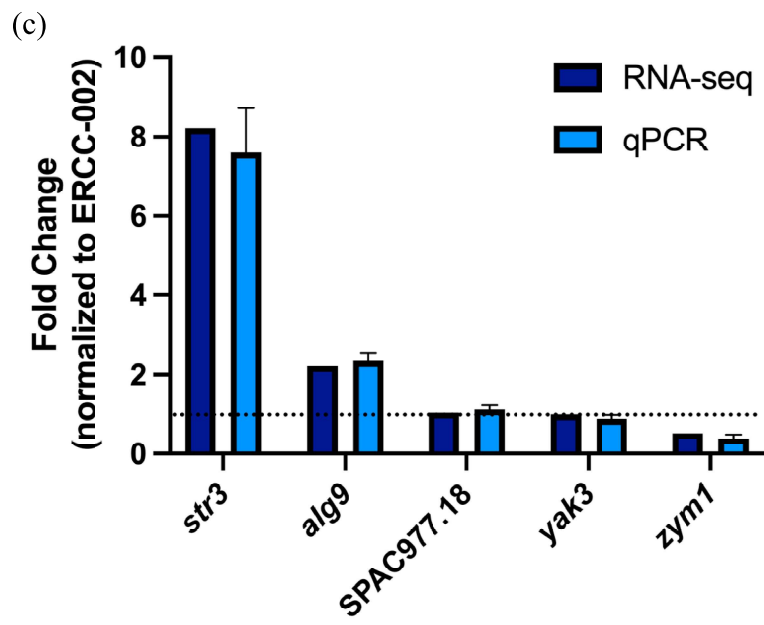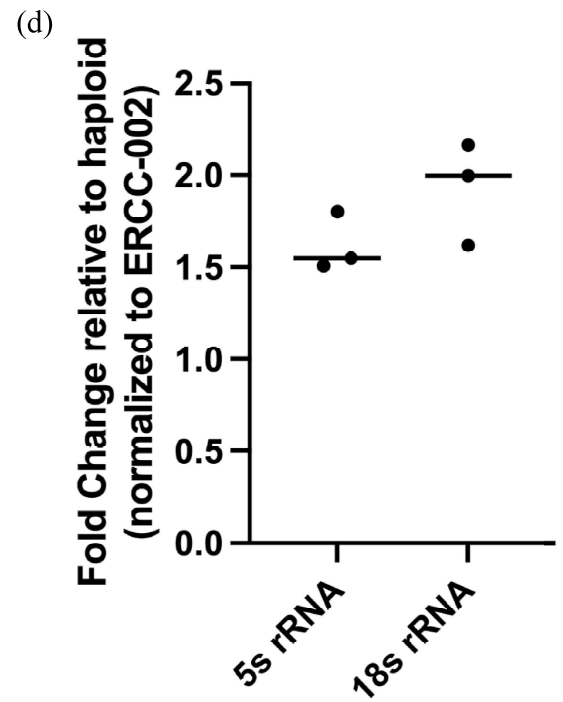

Supplement: jkae177_Supplementary_Data [file jkae177_supplementary_data.zip › Figure_S1_G3-2024-405152.pdf]

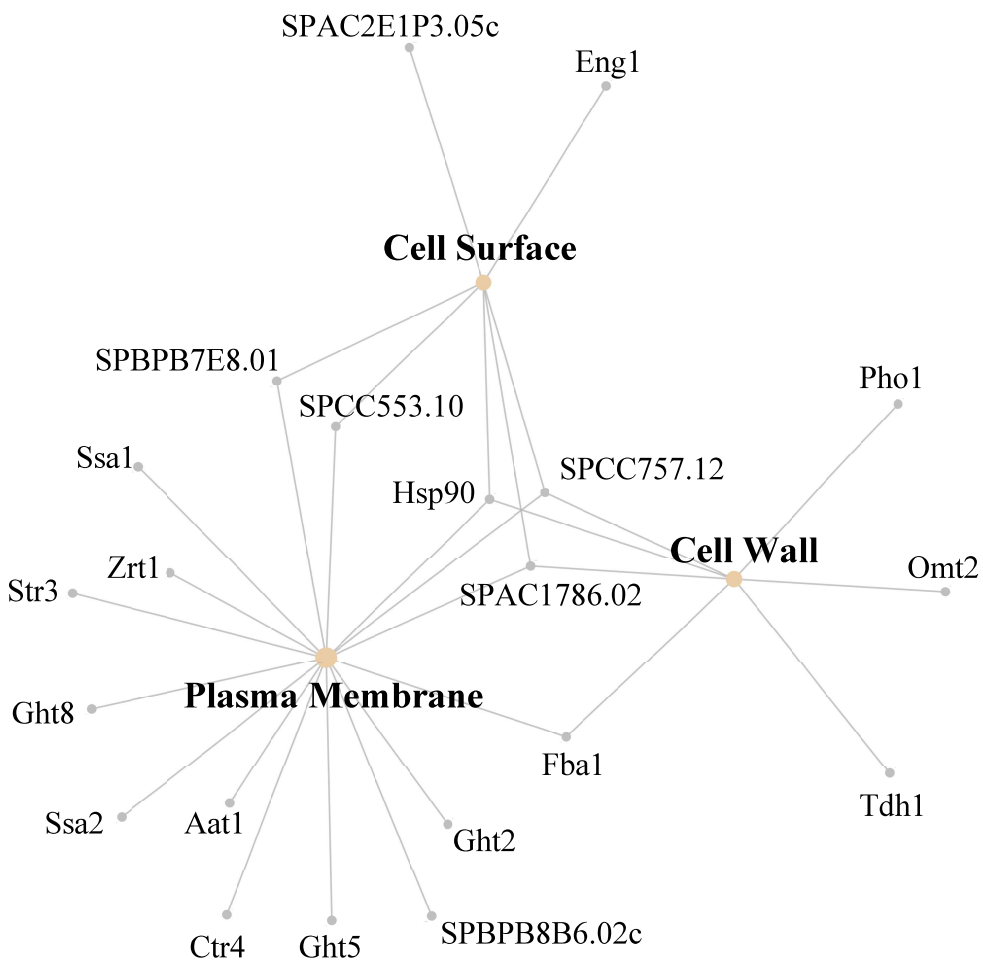

Supplement: jkae177_Supplementary_Data [file jkae177_supplementary_data.zip › Figure_S2_G3-2024-405152.pdf]
